# Impact of neo-adjuvant Sorafenib treatment on liver transplantation in HCC patients - a prospective, randomized, double-blind, phase III trial

**DOI:** 10.1186/s12885-015-1373-z

**Published:** 2015-05-11

**Authors:** Katrin Hoffmann, Tom Ganten, Daniel Gotthardtp, Boris Radeleff, Utz Settmacher, Otto Kollmar, Silvio Nadalin, Irini Karapanagiotou-Schenkel, Christof von Kalle, Dirk Jäger, Markus W Büchler, Peter Schemmer

**Affiliations:** 1 Department of General-, Visceral- and Transplantation-Surgery, Ruprecht-Karls-University, Im Neuenheimer Feld 110, 69120 Heidelberg, Germany; 2 Department of Internal Medicine, Ruprecht-Karls-University, Im Neuenheimer Feld 410, 69120 Heidelberg, Germany; 3 Department of Radiology, Ruprecht-Karls-University, Im Neuenheimer Feld 110, 69120 Heidelberg, Germany; 4 Department of General-, Visceral- and Vascular-Surgery, University Hospital, Erlanger Allee 101, 07747 Jena, Germany; 5 Department of General and Visceral Surgery, Georg-August-University, Robert-Koch-Str. 40, 37075 Göttingen, Germany; 6 Department of Surgery, University Hospital, Hoppe-Seyler-Straße 3, 72076 Tübingen, Germany; 7 National Centre of Tumour Diseases, Ruprecht-Karls-University, Im Neuenheimer Feld 460, 69120 Heidelberg, Germany; 8 Department of General- Visceral- and Transplantation Surgery, University of Heidelberg, Im Neuenheimer Feld 110, D-69120 Heidelberg, Germany

**Keywords:** Hepatocellular carcinoma, Liver transplantation, Sorafenib, Transarterial chemoembolization

## Abstract

**Background:**

Liver Transplantation (LT) is treatment of choice for patients with hepatocellular carcinoma (HCC) within MILAN Criteria. Tumour progression and subsequent dropout from waiting list have significant impact on the survival. Transarterial chemoembolization (TACE) controls tumour growth in the treated HCC nodule, however, the risk of tumour development in the untreated liver is increased by simultaneous release of neo-angiogenic factors. Due to its anti-angiogenic effects, Sorafenib delays the progression of HCC. Aim of this study was to determine whether combination of TACE and Sorafenib improves tumour control in HCC patients on waiting list for LT.

**Methods:**

Fifty patients were randomly assigned on a 1:1 ratio in double-blinded fashion at four centers in Germany and treated with TACE plus either Sorafenib (n = 24) or placebo (n = 26). The end of treatment was development of progressive disease according to mRECIST criteria or LT. The primary endpoint of the trial was the Time-to-Progression (TTP). Other efficacy endpoints were Tumour Response, Progression-free Survival (PFS), and Time-to-LT (TTLT).

**Results:**

The median time of treatment was 125 days with Sorafenib and 171 days with the placebo. Fourteen patients (seven from each group) developed tumour progression during the course of the study period. The Hazard Ratio of TTP was 1.106 (95% CI: 0.387, 3.162). The results of the Objective Response Rate, Disease Control Rate, PFS, and TTLT were comparable in both groups. The incidence of AEs was comparable in the placebo group (n = 23, 92%) and in the Sorafenib group (n = 23, 96%). Twelve patients (50%) on Sorafenib and four patients (16%) on placebo experienced severe treatment-related AEs.

**Conclusion:**

The TTP is similar after neo-adjuvant treatment with TACE and Sorafenib before LT compared to TACE and placebo. The Tumour Response, PFS, and TTLT were comparable. The safety profile of the Sorafenib group was similar to that of the placebo group.

**Trial registration:**

ISRCTN24081794

## Background

The age-adjusted incidence of hepatocellular carcinoma (HCC) has increased continuously over the past twenty years, making HCC to become one of the fastest growing causes of cancer-related deaths in the United States and Europe [1]. The implementation of effective surveillance programs for patients with Hepatitis B and C infection has significantly augmented the proportion of patients who are diagnosed at an early stage of the disease. Currently, 30-40% of patients are amenable to curative treatment options and the numbers are expected to increase to 60% within the coming decade [2]. Liver Transplantation (LT) is the only therapy that simultaneously cures the tumour and the underlying liver disease and the MILAN Criteria remains the benchmark for patient selection. Five-year survival rates of 60-70% have been achieved in high-volume centres with well-selected patients [3,4]. Unfortunately, there remains a great disparity between organ availability and demand; of all the patients on the waiting list for LT, less than one third actually undergo liver transplantations, while most of the enlisted patients simply drop out due to tumour progression [5]. Progression of the disease during the waiting time is associated with poorer transplant outcome [6]. Furthermore, the remaining, viable tumour in the explanted liver has been identified a risk factor for disease recurrence [7]. Although locoregional bridging therapies such as transarterial chemoembolization (TACE) are known to improve the survival rates of HCC candidates awaiting LT, their impact on tumour progression and dropout risk is still uncertain [8]. Complete response is seldom achieved via TACE and the presence of residual vital tumour is extremely difficult to evaluate. Nevertheless, locoregional therapies are recommended for bridging before LT [9]. Angiogenic factors are known to be released after locoregional therapy treatment and are believed to trigger tumour growth in untreated livers [10]. However, it might be possible to reduce these effects by using a treatment combination with an anti-angiogenic agent.

The oral multi-kinase inhibitor, Sorafenib, has shown significant efficacy in prolonging the Time-to-Progression for tumours in two large, Phase III trials and is the standard treatment for patients with advanced HCC [11,12]. Unfortunately, data on the use of Sorafenib in a neo-adjuvant setting before liver transplantation are rare [13]. In a Monte Carlo probabilistic sensitivity analysis, Vitale *et al.* showed that a neo-adjuvant therapy with Sorafenib before LT may have beneficial effects on the survival rates [14]. The strategy of using combined locoregional and anti-angiogenic therapies has been proven in *in vivo* cases. Xu *et al.* showed that the administration of Sorafenib in conjunction with radiofrequency ablation reduced the VEGF and HIF-1α levels, decreased micro-vessel density, and inhibited tumour growth [15]. However, based on the experience with other VEGF-targeted therapies in a peri-operative setting, concerns have been raised regarding the anti-angiogenic effect of Sorafenib [16]. Reports on the risk of post-transplantation complications are sparse and heterogenic [17,18]. Furthermore, it has been speculated that the toxic effects of Sorafenib may be prevalent in the presence of vascular and biliary anastomoses, and might increase the risk of leakage or thrombosis [14,17].

To test the hypothesis of the beneficial effects of a combined locoregional and VEGF-targeted therapy on tumour progression before LT, we initiated a prospective, multi-centre, placebo-controlled, randomized, double-blind trial that investigates the neo-adjuvant treatment course with TACE and Sorafenib in HCC patients awaiting LT who have been categorized according to the MILAN criteria [19]. Here, we report the results of fifty patients treated in this trial and demonstrate that the Time-to-Progression while on waiting list is independent of Sorafenib treatment.

## Methods

### Eligibility criteria

This multi-centre, randomized, placebo-controlled, double-blind, Phase III trial was performed in four centres in Germany [19]. The study was approved by the review boards and independent ethics committees of the participating institutions (EudraCT-Nr.: 2008-002269-29, ethics committee of Ruprecht-Karls-University Heidelberg, Medical Faculty Friedrich-Schiller-University Jena, University Medicine Göttingen and Medical Faculty Eberhard-Karls-University Tübingen). The trial was done in accordance with the International Conference on Harmonization Good Clinical Practice Guidelines, the Declaration of Helsinki, and the applicable local regulatory requirements and laws [20].

Patients over 18 years of age who had been diagnosed with hepatocellular carcinoma according to the MILAN Criteria were eligible, provided that they were diagnosed according to the guidelines of the European Association for the Study of Liver Disease (EASLD) and were found to be suitable for liver transplantation. All patients had measurable disease parameters that had been classified according to mRECIST (modified Response Evaluation Criteria in Solid Tumours) with no evidence of radiologically definable major vascular invasion or extrahepatic metastases, a Karnofsky index greater than 80%, adequate liver function with bilirubin content of <3 mg/dl, a prothrombin time that was less than 1.5 times higher than the upper limit of the normal range, and adequate renal and haematological function, as well as, a negative pregnancy test . All patients provided written informed consent.

The exclusion criteria were: prior systemic, anticancer therapy or local tumour therapy (i.e. LITT; PEI, cryotherapy, RFA, TACE), thrombotic or embolic events (including transient ischemic attacks within six months before study treatment), a haemorrhage/bleeding event of Grade III within four weeks of first dose of the study drug, any reported cardiovascular disease such as myocardial infarction six months prior to the start of trial, chronic heart failure (revised NYHA Grade III-IV) or unstable coronary artery disease, and uncontrolled hypertension despite optimal medical management. Patients with uncontrolled infections and HIV-seropositive patients were also excluded.

### Study treatment and evaluation of adverse events

Patients were treated according to the HeiLivCa study protocol with either TACE plus Sorafenib (400 mg bid, orally) or TACE plus placebo until progression of disease was observed or liver transplantation was performed [19,21]. Dose reductions of the study medication were allowed in patients with clinically significant toxicities. The study medication was interrupted three days before and continued three days after each TACE. TACE was performed using carboplatin as a chemotherapeutic drug and Lipiodol was used as the embolizing agent, as previously described [22]. TACE was performed every four weeks until complete devascularisation of the treated nodule. Computed Tomography or Magnetic Resonance Imaging was done four weeks after each TACE, followed by TACE every eight weeks until tumour progression or LT. Medical history, physical examination, assessment of the performance status, adverse events, and biochemical and haematological parameters were carried out at baseline of two weeks after the start of study treatment and then every four weeks during the course of the trial. Adverse events were graded according to the National Cancer Institute Common Terminology Criteria for Adverse Events (NCI-CTCAE), Version 3 [23]. LT was performed as previously described [6]. Routine immuno-suppression included an initial, intraoperative, induction dose of prednisolone, followed by CNI-based immunosuppression with cyclosporine A or tacrolimus with MMF or prednisolone.

### Statistical considerations

The primary endpoint of the study was the Time-to-Progression (TTP) while on the waiting list, assessed by the mRECIST Criteria. Other efficacy endpoints were Tumour Response, Progression-free Survival (PFS), and the Time-to-LT. The ORR (Objective Response Rate) was defined as either Complete Response (CR) or Partial Response (PR). Patients with insufficient data for tumour assessment (e.g., no baseline or follow-up assessments) were considered as “Not meeting ORR criteria”. DCR (Disease Control Rate) was defined as CR, PR, or SD (Stable Disease). TTP was defined as the time between the first dose of the study medication and the first documentation of tumour progression. For patients with no documented tumour progression before the study cut-off point or for those who dropped out of the trial, the Censoring Date was defined as the last date on which the progression status was adequately assessed. PFS was defined as the time between the date of the first dose of the study medication and the date of the first indication of disease progression or death due to any cause, provided that the death occurred before tumour progression was documented. Patients without progression or death were censored on the date of the last tumour assessment during the study. The Time-to-LT was defined as the time from the date of first administration of the study medication to the date of LT.

The sample size calculation was based on the detection of significant differences in TTP, assuming that median TTP was 4.5 months for the placebo-arm and 7.5 months for the sorafenib-arm (delta of 3 months similar to delta in SHARP study as presented at ASCO 2007). Presupposed exponential survival curves, constant monthly hazard rates, an accrual period of 24 months and a total follow-up of 33 (=24 + 9) months, and testing for the above-mentioned difference at an overall one-sided significance level of 0.05 and power of 0.875 a total of 136 patients were required. From the experience gained at the surgery department it was anticipated that about 50% of the patients will drop-out after randomization. These patients do not contribute any information to the primary endpoint. In order to accommodate for a maximum drop-out rate of 50% the total sample size was therefore increased to 208 (104 per treatment group). Patients were randomly assigned on a 1:1 ratio in a blinded fashion to the sorafenib or placebo. A central computer-generated block-randomized list was prepared by an independent biostatistician of the KKS in Heidelberg and provided to the Pharmacy at University Hospital Heidelberg.

According to the study protocol, standard methods for survival analysis were to be used in the analysis of Time-to-Event endpoints, including Kaplan-Meier estimates of the survivor functions, Greenwood’s formula for estimating the standard error of event rates, the Cox Proportional Hazards Model, and the log-rank test for comparing survival curves. However, the HeiLivCa trial was stopped prematurely after the inclusion of fifty patients and, for this reason, any confirmatory statistical analysis was unattainable. Therefore, the statistical analysis actually performed was in a strictly exploratory and mainly descriptive manner, and differed from the analyses outlined in the study protocol. Due to the presence of competing risk factors in regards to TTP, PFS, and the Time-to-LT, the Kaplan-Meier estimator could not be used and, therefore, an analysis of the Competing Risk data was performed using the SAS macro % Cumulative Incidence Functions (CIF) [24]. The macro implements used for estimating Cumulative Incidence Functions in this study were appropriate for nonparametric methods. The cumulative incidence for a particular cause of failure is the probability of experiencing this cause of failure until time t, in the presence of all the other possible causes. The estimates of the CIF for each treatment group from the aforementioned Time-to-Event endpoints, the Standard Errors, and the 95% Confidence Intervals were displayed. Additionally, the test for equality of the CIF among the various treatment groups was performed using Gray’s method [25]. All data collected in regards to assessing the safety and efficacy of the trial are reported in the summary presentations, listings, or both. All statistical tests used in the exploratory analyses were two-tailed. The significance level was 0.05 and was calculated using SAS Version 9.3. The end of the study was defined when either the informed consent was withdrawn or the presence of disease progression or LT was reported.

### Evaluation of response

All patients had chest and abdominal imaging (Computed Tomography using Siemens Somatom Definition and/or Magnetic Resonance Imaging using Siemens, Symphony, 1.5 t) before the initiation of the Sorafenib therapy. Imaging was repeated during the trial as described in the study protocol to evaluate radiographic response within the tumour; this response was then classified using the mRECIST criteria. Densitometric measurement of intra-tumoural enhancement was performed using a modification of the Choi Criteria to evaluate the effect of the treatment course on tumour viability. Briefly, using axial source Computed Tomography images (3 mm thickness), a three-dimensional image of the liver was reconstructed with an imaging workstation (TeraRecon, USA). The largest dimensions of the HCC were outlined and a density measurement in Hounsfield units (HU) was obtained. The percental change in HU after treatment was recorded.

### Role of the sponsor

The study was an investigator-initiated trial (IIT) that was designed by the principal investigators, Peter Schemmer and Katrin Hoffmann. All logistical aspects of the study were managed by the study sponsor, namely, the University of Heidelberg. Data were collected by the study sponsor and all authors, and all the aforementioned parties had full access to the study data. The corresponding author had the final responsibility of submitting the manuscript for publication. The study was supported by Bayer Healthcare GmbH, Leverkusen, Germany.

## Results

### Patients

**Figure 1:**
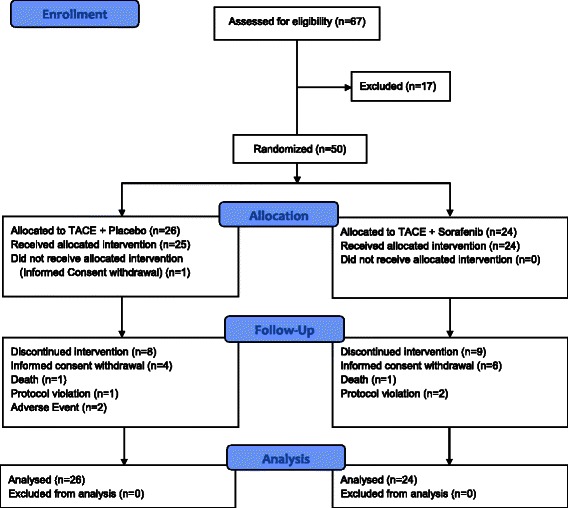
Consort flow diagram.

Of the sixty-seven patients who were screened, a total of fifty patients were treated within this study. One patient from the placebo group eventually withdrew the informed consent. This patient did not receive any study treatment and, as such, was excluded from the safety analysis. On the other hand, all fifty randomized patients were included in the efficacy analysis. Ten patients withdrew their informed consent (n = 6 from the Sorafenib group and n = 4 from the placebo group) due to compliance issues, two patients died (one from each group) during the study (one from a motorcycle accident and the other from general status impairment), three patients were excluded from analysis due to protocol violation, and two patients from the placebo group discontinued participation due to adverse events (a hip fracture and the development of oesophageal cancer) (Figure 1). The baseline characteristics of the study patients are displayed in Table 1. The median age of the participants was 58 years (range: 43 to 69 years) and this was representative of 45 males (90.0%) and 5 female patients (10.0%). A tumour histology obtained by biopsy was available for 14 patients (28.0%). No patient had metastatic disease and all were found to be within the MILAN Criteria (Table 1).

**Table 1:** **Baseline characteristics of study patients**

	TACE + Sorafenib		TACE + Placebo	
	n = 24		n = 26	
Age (years) median (min-max)	58.5	(44.0-66.0)	58.0	(43.0-69.0)
BMI (kg/m^2^) median (min-max)	27.5	(20.2-40.6)	26.3	(21.0-37.9)
Biopsy proven n (%)				
yes	8	(33.3%)	6	(23.1%)
no	16	(66.7%)	20	(76.9%)
Child pugh stage n (%)				
A	14	(58.3%)	20	(76.9%)
B	9	(37.5%)	6	(23.1%)
C	1	(4.2%)	-	
Karnofsky index n (%)				
80	6	(25.0%)	4	(15.4%)
90	10	(41.6%)	16	(61.5%)
100	8	(33.3%)	6	(23.1%)
Underlying liver disease n (%)				
Viral hepatitis				
B	3	(12.5%)	3	(11.5%)
C	11	(45.8%)	7	(26.9%)
alcoholic	7	(29.1%)	11	(42.3%)
other	3	(12.5%)	5	(19.2%)
MELD score at listing median (min-max)				
labMELD score	11	(6–15)	11	(6–37)
exceptional MELD score	27.5	(27.5-40.1)	25.2	(21.9-40.1)
AFP (IU/ml) median (min-max)				
start of study	13.2	(1.2 – 2.961)	9.8	(1.3 – 225)
end of study	6.2	(1.0 – 1.788)	9.4	(2.0 – 362)

### Treatment and adverse events

The median number of days on Sorafenib was 125 (range: 1 to 380 days) and the median duration of treatment with the placebo was 171 (range: 1 to 366 days). TACE was performed in a median number of two in patients of the Sorafenib group (range: 0 to 4) and three in patients of the placebo group (range: 2 to 4). No TACE-associated complications were observed. The majority of the patients (46 of 50) experienced at least one AE. The incidence of AEs was slightly lower in the placebo group (92%, 23 of 26 patients) than in the Sorafenib group (96%, 23 of 24 patients). The most frequent, treatment-related AEs were thrombocytopenia, diarrhea, and hand-foot-skin reactions. A total of 22 patients (92%) in the Sorafenib group and 21 patients (84%) in the placebo group experienced at least one treatment-related AE. Twelve patients (50%) on Sorafenib and four patients (16%) on the placebo experienced “Severe” treatment-related adverse events (CTCAE Grade III or IV), while a total of six patients (three patients from each group) experienced “serious” treatment-related adverse events (Table 2). Eight patients (n = 2 from the placebo group and n = 6 from the Sorafenib group) had dose reductions or temporary discontinuations due to treatment-related adverse events. Seven patients (n = 1 (4.0%) from the placebo group and n = 6 (25%) from the Sorafenib group) discontinued using the study drug due to treatment-related AE. Even though four patients (two from each group) died during the observation period, it must be said that there were no treatment-related deaths in general. The safety profile of Child A and Child B patients was almost similar.

**Table 2:** **Treatment related adverse events classified according to CTC-AEv3.0 Frequencies and 95% (two-sided) confidence intervals according to Pearson-Clopper**

	TACE + Sorafenib			TACE + Placebo		
	N = 24			N = 25		
	n	%	95% CI	n	%	95% CI
Patients with at least one AE	22	91.7	73.0 - 99.0	21	84.0	63.9 - 95.5
Blood/lymphatic disorders						
ucopenia	10	41.7	22.1 - 63.4	3	12.0	2.5 - 31.2
thrombocytopenia	13	54.2	32.8 - 74.4	14	56.0	34.9 - 75.6
Gastrointestinal disorders						
diarrhoea	9	37.5	18.8 - 59.4	3	12.0	2.5 - 31.2
nausea	3	12.5	2.7 - 32.4	2	8.0	1.0 - 26.0
General disorders						
fatigue	5	20.8	7.1 - 42.2	5	20.8	6.8 - 40.7
weight loss	1	4.2	0.1 - 21.1	-		
Hepatobiliary disorders						
hyperbilirubinaemia	3	12.5	2.7 - 32.4	3	12.0	2.5 - 31.2
cholangitis	1	4.2	0.1 - 21.1	-		
Dermatologic disorders						
hand-foot-syndrome	7	29.2	12.6 - 51.1	1	4.0	0.1 - 20.4
alopecia	1	4.2	0.1 - 21.1	-		
Sever AE	12	50.0	29.1 - 70.9	4	16.0	4.5 - 36.1
SAE (CTC-AEv3.0 GRADE 3/4)	3	12.5	2.7 - 32.4	3	12.5	2.5 - 31.2
Study Drug discontinued due to AE	6	25.0	9.8 - 46.7	1	4.0	0.1 - 20.4
Dose reduced due to AE	6	25.0	9.8 - 46.7	2	8.0	1.0 - 26.0
AE resulting in death	-			-		

### Effect on outcome measures

**Figure 2:**
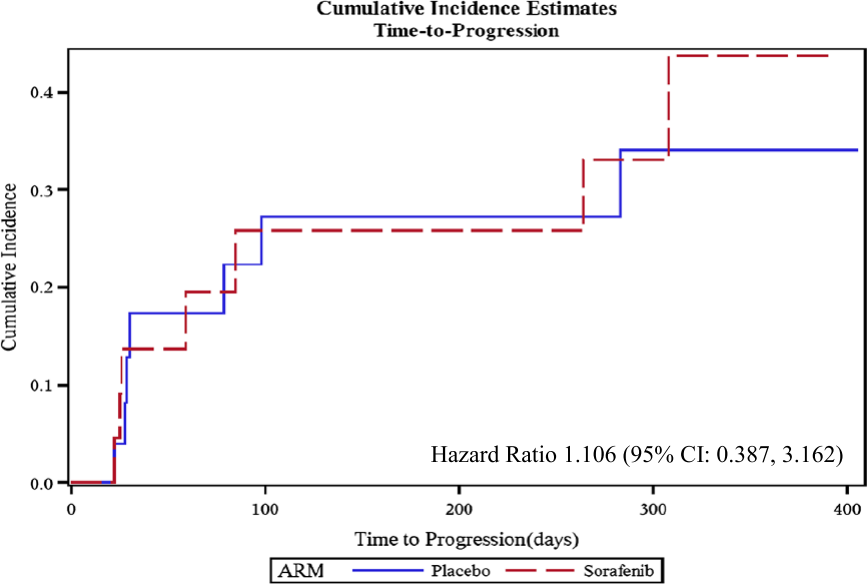
Estimated Cumulative Incidence Functions for the Time-to-Progression for the two treatment groups.

**Figure 3:**
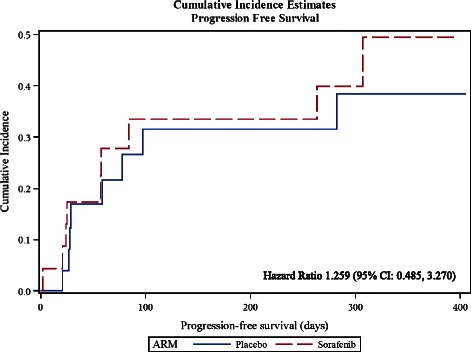
Estimated Cumulative Incidence Functions for the Progression-free Survival Rate for the two treatment groups.

There were a total of fourteen patients (seven patients from each group) who experienced tumour progression during the course of the study. The median TTP was 71 days (range: 1 to 394 days) in the Sorafenib group and 85 days (range: 1 to 405 days) in the placebo group. The estimates of the CIF of TTP, the Standard Errors, and the 95% Confidence Intervals were similar in the two treatment groups. The Hazard Ratio was 1.106 (95% CI: 0.387, 3.162) (Figure 2). The PFS was comparable in the both groups and in this case, the Hazard Ratio was 1.259 (95% CI: 0.485, 3.270) (Figure 3).

The study ended when there was withdrawal of consent and/or the occurrence of disease progression or LT. During the last study visit, patients of the Sorafenib group showed CR (n = 1, 4.3%), PR (n = 4, 17.4%), SD (n = 11, 47.8%), and PD (n = 7, 30.4%) in comparison to patients of the placebo group CR (n = 0), PR (n = 7, 26.9%), SD (n = 12, 46.2%), and PD (n = 7, 26.9%). The Objective Response Rate (CR + PR) was 20.8% (95% CI: 7.1 - 42.2) in the Sorafenib group and 26.9% (95% CI: 11.6 - 47.8) in the placebo group. The Disease Control Rate (CR + PR + SD) was 66.7% (95% CI: 44.7 - 84.4) in the Sorafenib group and 73.1% (95% CI: 52.2 - 88.4) in the placebo group. Mean change in AFP from baseline to last visit was −76.7 (SD = 267.5, median = −1.9) in the TACE plus Sorafenib group and 6.5 (SD = 64.2, median = −1.7) in the TACE plus Placebo group. Considering mean changes between baseline and last visit, differences were most pronounced in the TACE plus Sorafenib group.

### Liver transplantation

**Figure 4:**
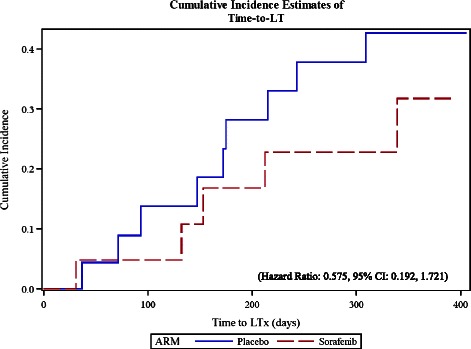
Estimated Cumulative Incidence Functions for the Time-to-liver transplantation for the two treatment groups.

Due to the design of the trial, additional information on LT has been obtained when the trial was finished. In total, transplantation was performed in seventeen patients who were actively involved in the trial at time of the surgery and had no evidence of disease progression. In sixteen of the patients, LT was performed in a modified Piggy-back technique with organs from cadaveric donors [6]. One patient underwent an extended, right-lobe transplantation using a graft from a cadaveric donor. LT was performed in five patients (22.7%) from the Sorafenib group and in twelve patients (46.2%) from the placebo group. The median TTLT was 153 days in the Sorafenib group (range: 31 to 339 days) and 174 days (range: 37 to 315 days) in the placebo group (Hazard Ratio: 0.575, 95% CI: 0.192, 1.721) (Figure 4).

The median operative times, blood loss and length of the participants’ hospital stay were similar in patients from the Sorafenib group and patients from the placebo group (*p* = 0.02; *p* = 0.25 and *p* = 0.98). The overall 30-day morbidity rate was 35%. Surgical re-intervention was necessary in two patients from the placebo group due to the occurrence of Ogilvie syndrome in one case and bile duct leakage in the other. ERC due to bile duct stenosis was necessary in two patients (one from each group). Radiological re-intervention was necessary in two patients (n = 1 from the Sorafenib group which was caused by coiling of the splenic artery due to steal phenomenon and n = 1 from the placebo group due to an infected bilioma). No complications of delayed wound-healing, bowel dehiscence, or incisional hernia were observed in the Sorafenib group. Acute rejection (Banff score 3 to 7) occurred in three patients (n = 1 from the Sorafenib group and n = 2 from the placebo group).

Nine patients remained alive after a median follow-up of 320 days. One patient from the placebo group died within 30 days after LT due to cardiac decompensating. A total of five patients died during the follow-up period (n = 1 from cerebral oedema at four months after LT, n = 1 from myocardial infarction, n = 1 from sepsis after colonic ischemia at six months after LT, n = 1 from general sepsis, and n = 1 from multiple organ failure at 24 months after LT). Re-transplantation was performed in two patients (n = 1 was performed 23 days after LT and was due to primary non-function; n = 1 was performed three days after LT and was due to acute graft ischemia). Two transplanted patients who had been treated with the placebo experienced recurrence of the disease with pulmonary metastases within the first year of LT and subsequently died. Currently, there are no signs of recurrence in all other patients still alive after LT.

## Discussion

Without potentially curative liver transplantation, hepatocellular carcinoma has a dismal prognosis. The concept of locoregional treatment for bridging while awaiting LT has been proven to be effective within the last decade and excellent outcomes have been reported for the TACE treatment option [26,27]. A meta-analysis clearly showed the beneficial survival effect of TACE in comparison to the results obtained from untreated cases [28]. In recent years there was considerable effort made to find the optimal therapeutic regimen of transarterial chemoembolization. Most centers now use drug eluting beads for their TACE procedures and doxorubicin as chemotherapeutic drug [29].

However, the effectiveness of TACE is hampered by tumour progression during the waiting time and subsequent drop-out rates of up to 53% [30,31]. Recent evidence suggests that neo-angiogenic reactions are induced after TACE, which in turn, potentially enhances the tumour growth of untreated nodules or accelerates the development of de-novo tumours within the cirrhotic liver [10]. Furthermore, a high increment of VEGF after TACE has been recently identified as an independent, negative prognostic factor for both the progression-free and overall survival rates [32,33]. Sorafenib has direct inhibitory effects on angiogenesis and cell proliferation in HCC; and in two large, Phase III, randomized, controlled trials with demographically different study populations of patients with advanced HCC, Sorafenib has been shown to significantly improve the time to progression [12,34]. The HeiLivCa trial was designed to capture the potentially beneficial effects of a combined locoregional and systemic, molecular-targeted treatment course for HCC patients awaiting LT. The TTP was selected as the primary endpoint of the study because it is an efficient means of monitoring the effects associated with the stabilization of the disease for a clinically relevant period of time.

Previously, the use of Sorafenib in a post-transplantation setting was controversially discussed because of its toxicity. This was especially true when the occurrence of Grade III - IV adverse events in the majority of patients ultimately resulted in permanent dose reduction or treatment discontinuation [35-37]. However, the overall experience with Sorafenib in HCC patients awaiting LT is actually even bleaker. Concerns regarding the potential negative side effects of Sorafenib have been raised [17,18,38-40]. Nevertheless, the performance of the trial was encouraged by emerging reports about a near absence of liver toxicity or treatment-related deaths [12,34].

There are three points that are of major interest when discussing a neo-adjuvant Sorafenib treatment course before LT. The first is the safety when it is used in combination with locoregional treatment options. Data from studies that investigated the combined use of TACE and Sorafenib in patients with advanced HCC suggests that this treatment option has an acceptable safety profile [41]. In the HeiLivCa trial, 92% of patients in the Sorafenib group experienced at least one treatment-related adverse event. Of those, grade III or IV adverse events occurred in 50% of patients. The major side effects observed in this study were consistent with those reported in two previously conducted randomised controlled trials and a pooled safety analysis of a Sorafenib monotherapy trial for patients with advanced HCC [12,34,42]. The most frequent adverse events in our trial were thrombocytopenia, diarrhea, and hand-foot-skin reactions, however, these events occurred less frequently in comparison to the safety reports from previous Sorafenib monotherapy trials. Furthermore, despite overlapping co-morbidities in Child-Pugh class B patients, the treatment was equally well tolerated in Child-Pugh class A and B patients. In this trial, only 24% of patients in the Sorafenib group had dose reductions or temporary discontinuations due to treatment-related adverse events; a figure that is significantly lower than those values reported in other trials [17,18]. The occurrence of fewer dose adjustments in more recent trials could be attributed to the fact that physicians have gained more experience in handling molecular-targeted therapies, adverse events are monitored more aggressively, and the management strategies for side effects have been optimised over time [43]. There has been growing concern about the increasing numbers of TACE-associated complications when the therapy is used in combination with an anti-angiogenic drug. In light of this, we decided to interrupt drug treatment three days before and after each TACE for this study. As a result of this strategy, no TACE-associated or bleeding complications were observed.

The second point of interest on this topic is the effect of Sorafenib on tumour growth during the waiting time. In this study, the TTP remained constant with the combined TACE plus Sorafenib therapy and the median TTP was similar in both treatment groups. However, TTP has previously been challenged as a surrogate endpoint in trials for advanced HCC because of the inconsistencies between the OS and the TTP that have been detected in other studies. ORR is currently being revisited as an alternative endpoint after the introduction of modified RECIST assessment in HCC. In our trial, the ORR and the Disease Control Rate were also comparable in groups. Truesdale *et al.* reported that there were no dropouts due to disease progression in the Sorafenib group of their study [17]. Kulik *et al.* reported the occurrence of disease progression during the trial in one patient under Sorafenib and one patient of the control group [40]. Frenette *et al.* gave no information on disease progression in their patients, however, this could have been influenced by the fact that both their study setting was different and that 93% of their patients were beyond the MILAN criteria [18]. Nevertheless, all three groups reported that Sorafenib had no general impact on overall survival rates after LT [17,18,40]. A potential explanation for a failure of Sorafenib treatment lies within the individual tumor biology of each patient. HCC can be seen as a prototypical therapy-resistant tumour [44-46]. Most recently analysis of therapy-naïve HCC tissue has shown that baseline tissue expression of pERK and VEGFR-2, both key molecules in the cancerogenic pathway of HCCs, is inversely correlated with the clinical outcome in advanced HCCs treated with Sorafenib [47]. Furthermore, a pooled shRNA screen conducted to identify target genes whose inhibition increases the therapeutic efficacy of Sorafenib identified MAPK14-dependent activation as a key mechanism of Sorafenib resistance in mouse and human liver cancer [48]. However, the incidence of severe adverse events in the Sorafenib treated patients and the combination with the lack of an impact on the study end-points discourage in our view the use of the drug in combination with TACE.

Lastly, the interaction of Sorafenib with the transplantation setting is of particular interest for transplant surgeons. Previous research with molecular-targeted therapies in a pre-operative setting has raised concerns about the risk of wound-healing complications, haemorrhage, and cardiac events. The current study did not identify an overall increased risk of delayed wound-healing, bowel perforation, or incisional hernia. The intraoperative blood loss was not higher in the Sorafenib group and this was in line with the data from Frenette’s research [18]. On the other hand, Kulik *et al.* and Truesdale *et al.* described both a potentially increased risk for biliary complications and a higher rate of rejection after neo-adjuvant Sorafenib use [17, 40]. However, the survival rates of two aforementioned trials were the same in both the Sorafenib and the placebo groups.

We are well aware of the limitations of the present study. To our knowledge, this is the largest cohort reported to date. This study was designed under the assumption that Sorafenib provides a 40% reduction in the hazard ratio for TTP. However, the results showed no treatment difference between Sorafenib and placebo. The observed hazard ratio in TTP for Sorafenib compared to placebo was 1.106, 95% CI of 0.387 to 3.162, indicating a slight disadvantage for Sorafenib. Based on the data shown above, we calculated the conditional power according to Andersen [49]. Even if the accrual would be extended for another four years the conditional power (based on the initial assumptions regarding treatment effect) would be less than 10%. Consequently, we stopped the study due to futility. With that being said, we opted to report the results of the fifty patients treated in the trial because of the clinical relevance. Due to the low patient numbers, any confirmatory statistical analysis was unattainable inappropriate, therefore, the statistical analysis that was actually performed was in a strictly exploratory and mainly descriptive manner. It should also be emphasized that it is highly probable that a type 2 error, because of the small sample size, would make the differences in complications after LT difficult to detect.

## Conclusion

In conclusion, we believe that this trial gives no evidence on the indication of Sorafenib for HCC before LT. The Time-to-Progression, the Objective Response Rates, and the Disease Control Rates remained similar after the administration of the combined neo-adjuvant treatment with TACE and Sorafenib to HCC patients in our trial setting. In combination with the increased incidence of adverse events a recommendation for neo-adjuvant treatment with Sorafenib and TACE cannot be given.

## Abbreviations

CIF: Cumulative Incidence Functions
CR: Complete Response
CTCAE: Common Terminology Criteria for Adverse Events
DCR: Disease Control Rate
EASLD: European Association for the Study of Liver Disease
HCC: Hepatocellular Carcinoma
IIT: Investigator-initiated Trial
LT: Liver Transplantation
mRECIST: Modified Response Evaluation Criteria in Solid Tumours
ORR: Objective Response Rate
PFS: Progression-free Survival
PR: Partial Response
SD: Stable Disease
TACE: Transarterial chemoembolization
TTP: Time-to-Progression
TTLT: Time-to-Liver-Transplantation
